# Automatic Classification of Protein Functions From the Literature

**DOI:** 10.1002/cfg.241

**Published:** 2003-02

**Authors:** Christian Blaschke, Alfonso Valencia

**Affiliations:** Protein Design Group National Center for Biotechnology CNB-CSIC Cantoblanco Madrid E-28049 Spain


**Comparative and Functional Genomics**
*Comp Funct Genom* 2003; **4**: 75–79.
Published online in Wiley InterScience (www.interscience.wiley.com). **DOI:** 10.1002/cfg.241

**Conference Review**

### Automatic classification of protein functions from the literature

Christian Blaschke and Alfonso Valencia*
*Protein Design Group, CNB-CSIC, Madrid, Spain*

**Correspondence to:*
*Alfonso Valencia, Protein Design Group, National Center for Biotechnology, CNB-CSIC, Cantoblanco, Madrid E-28049, Spain.*
*E-mail: valencia@cnb.uam.es*

Received: 6 November 2002
Accepted: 29 November 2002

Key activities in genomics and proteomics are related to the use of classifications (ontologies). The three main activities are:

1. Annotation of gene function after large-scale genome sequencing projects or in protein databases.
2. Annotation of the possible function common to sets of genes with similar expression profiles (determined after DNA array experiments).
3. Annotation of the characteristic functions of groups of proteins, protein complexes and biological pathways (determined by proteomics approaches).

The process of annotation requires the work of human experts who, by reading the available literature and using their expert knowledge, are able to generate abstractions regarding the common function of genes and proteins.

This process is extremely demanding and time consuming and usually has to be repeated many times (e.g. for every different clustering process applied to the same DNA array experiment). In addition, the pointers to the evidence used by the annotators must be recorded (the experimental evidence should be linked to the annotations).

The GO ontology [2] has become a *de facto* standard that helps to solve some of these problems, providing fast access to the annotations of various functional characteristics (metabolic, chemistry, localization). However, a number of the requirements described above are still not addressed, including the need to keep pointers to the underlying information up to date and the need to provide reproducible evidence (e.g. why this group of sequences was assigned to that function). Furthermore, the annotation of functions at levels that differ from the ones represented in GO, or genomes not yet included, also remains to be solved.

Statistical information extraction is a field with a long history that has only recently been applied to biology. One of the best-known applications has been integrated into PubMed to enhance the search functionality for Medline queries [21,22]. Special applications were dedicated to knowledge base construction [7,14], analysis of DNA microarray data [13,19] and improvements of the search capabilities in document collections [16].

### Systems for the annotation of groups of genes (gene products)

These methods were developed for the functional annotation of protein families, results of DNA array expression experiments, and data from proteomics.

Copyright © 2003 John Wiley & Sons, Ltd.

They are based on statistical information extraction techniques that basically use term frequencies and their distribution in the text to characterize a given text corpus. Here the criterion is whether a term appears more frequently in a given document set than in other comparable documents. Terms will have high (statistical significance) values if they are frequent in the documents under consideration and not frequent in the rest of the groups.

These systems extract (from a set of documents) specific terms that are related to a number of genes or proteins of interest, detect highly informative sentences in the text and rank the documents according to their information content, and thus reduce the number of documents that have to be reviewed for a set of genes or proteins.

### Specific applications related to the annotation of protein families and gene clusters

This basic engine has been adapted for the analysis of protein families [1,3], clusters of genes from DNA array experiments [4,15] and protein complexes (not published), e.g. for functionally related proteins implicated in vacuolar ATP synthesis, the system extracts terms such as *vacuolar acidification, vacuolar ATPase* or *proton-translocating ATPase*; for proteins related to the cell cycle, terms such as *microtubule, spindle pole* or *kinesin-related* (showing the strong relationship between cell division and the cytoskeleton) are extracted.

The main result of expression array experiments is the discovery of sets of genes with similar expression patterns (expression-based gene clusters). The underlying assumption is that these clusters are related by their participation in common biological processes. These similarities are reflected in the individual publications about the genes in a given cluster and can be detected by our methods.

We analysed gene expression data, including the experiments in yeast published by Eisen *et al.* [9]. These experiments monitored the expression of yeast cells in 79 separate experiments, including diauxic shift, mitotic cell cycle, sporulation, and temperature and reducing shocks. The system was applied to the 254 genes that showed significant changes in gene expression, corresponding to 10 clusters. Figure 1 shows part of the results generated by the system.

The results obtained for a cluster containing genes related to DNA replication initiation and entrance into the cell cycle, including cell division control (CDC) genes such as cdc47 and cdc54, genes related to minichromosome maintenance (mcm2 and mcm3), and dbf2, a protein kinase related to cell division, illustrate the quality of the terms extracted by the system. The terms extracted were related to minichromosome maintenance (mcm2, mcm3, mcm4, mcm5, cdc46, mcm proteins, mcm family, mcm genes, minichromosome maintenance, maintenance mcm, mis5, chromosome loss), DNA synthesis (licensing factor, replicate, replication licensing, replication origins, autonomously replicating, DNA replication, DNA synthesis, S-phase), phosphorylation (protein kinase, dbf2, phosphorylate) and cell cycle (cdc46, cdc47, cdc21, cdc54, cell cycle).

### Problems with gene and protein names

The main challenge is still the correct identification of all the pertinent text, i.e. all the documents (abstracts) related to the genes (or gene products) under analysis. Using current techniques it is not difficult to identify the entities in the text (this is the part of the analysis called 'named entity recognition' in the context of information extraction) but, because of ambiguities in the names of biological objects and the absence of a strict nomenclature for gene and protein names, these entities are difficult to classify.

The problem is related to the different ways in which the same gene (or gene product) can be referred to in text. Our previous evaluation shows that even in well-annotated, manually curated databases a substantial number of records contain protein names that cannot be identified in the literature [5].

A number of groups have addressed this problem using different technologies [11,17,20] and Franzén *et al.* recently reported some improvements [10]. Still, no perfect solution is at hand. The problem is not only due to the number of ways in which gene and protein names can be written (e.g. IL6, IL-6 or IL 6, for interleukin 6); other issues are that names can be part of other names

Copyright © 2003 John Wiley & Sons, Ltd. 

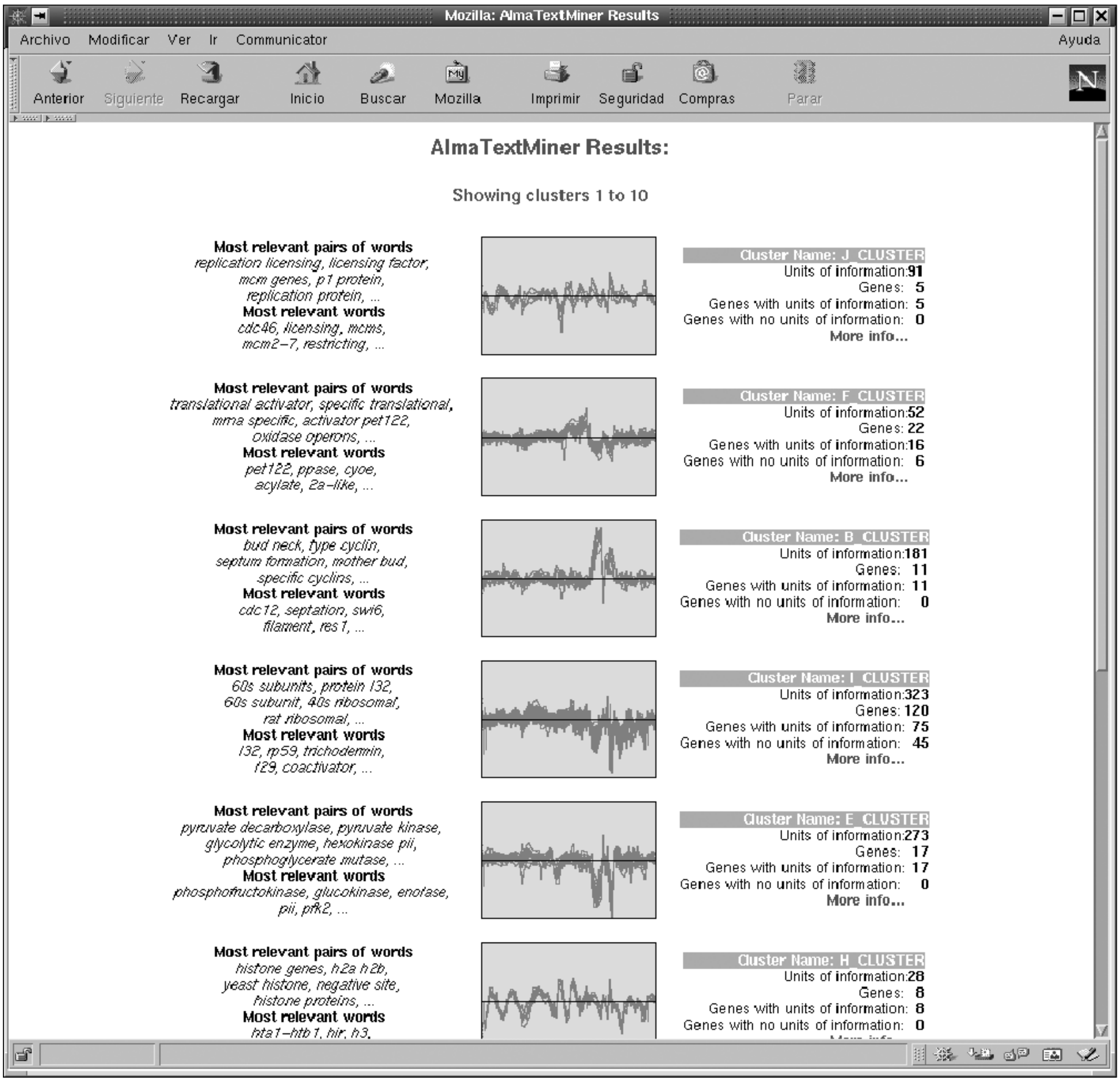

**Figure 1.** Results of an analysis of a DNA array expression experiment. The figure shows extracted keywords for each cluster (on the left) and the corresponding expression profiles (middle). This output was produced with an implementation of the methods described in the publications mentioned above by Alma Bioinformatica S.L. (**www.almabioinfo.com**) under the name AlmaTextMiner

(e.g. Cdc7 and Cdc7 protein kinase), that they can include non-protein name parts (e.g. RNA in RNA polymerase II), and that they can refer to classes of proteins rather than a specific protein (e.g. Fus3p and Kss1p are MAP kinases, CLN1 and CLN2 are $G_1$/S cyclins). Furthermore, a protein can be referred to by its specific name or by its general class name (cyclin B or 'this cyclin'), and gene and protein names can easily be confused with the names of other biological entities (e.g. drugs).

### Creation of ontologies for the specific annotation of protein function

Following the initial methodology for the extraction of information for the functional annotation of genes and proteins, we implemented a system that would detect similarities between genes based on the literature published about them [6]. These similarities were then used to cluster genes and to construct tree-like structures with genes at the

Copyright © 2003 John Wiley & Sons, Ltd.

leaves and concepts (terms) at the nodes of the tree, each one of them weighted by its statistical significance.

The system produces similar annotations to those given by GO, particularly for aspects related to biochemical function. Additionally, the terms and document links that justify the proposed relations are provided, to assist human experts in building the ontologies.

For example, the system associated a number of genes implicated in cell cycle regulation, e.g. $G_1$ and $G_2$ cyclins, the protein kinases cdc28 and cdc7, and DNA polymerases, because they all shared a number of significant terms (e.g. $G_1$ cyclin, cell cycle, s-phase) that indicate that they are implicated in similar processes. Figure 2 shows an example of the results for a number of genes that are all implicated in DNA repair in yeast.

```
        +----* pol30
  +-----+
  |     |    +---* ubc13
  |     +----+
  |          +---* mms2
 -+
  |          +----* rad18
  |     +----+
  |     |    |    +---* rad6
  |     |    +----+
  |     |         +---* ubc2
  +-----+
        |    +----* rev3
        +----+
             |    +----* rad30
             +----+
                  |    +---* rev7
                  +----+
                       +---* rev1
```

**Figure 2.** Part of the gene structure constructed by the system. These genes were clustered close together because of their common implication in DNA repair

The system includes facilities for: (a) the use of large repositories of published knowledge to enrich the information associated with the existing ontologies; (b) the automatic suggestion of the classification of new entities; and (c) the introduction of possible new pointers to the corresponding literature. The process has the advantage of producing annotations that can be directly related to the corresponding sentences and documents, providing additional words and concepts to supplement those provided in the human-driven ontologies.

The utility of automatic classification systems may be particularly relevant for database annotators during the construction of ontologies for new organisms. Normally, sequence similarity is used to assist in the classification of new genes and/or the extension of classifications to other organisms. Unfortunately, it is now clear that the operations of functional transfer based on sequence similarities tend to produce a large number of erroneous annotations [8], precluding the automatic construction of functional ontologies from the simple study of sequence relations. Therefore, the extension of the ontologies to new sequences requires extensive consultation of bibliographic information. This process can be facilitated and streamlined by applications such as the one presented here. For another recent publication addressing similar problems, see [18].

### Evaluation of information extraction systems

In the natural language processing (NLP) field there exists a long tradition of evaluating information extraction systems in public competitions (e.g. in the so-called *Message Understanding Conferences* held since the 1980s [12]). This requires a considerable organizational effort to define a common goal that is both tractable and of practical value (i.e. a problem that is of real world interest and that reflects, at least to some extent, to what point the current techniques are 'useful'), and to prepare annotated training and test corpora, and to evaluate the results.

No competition like these has yet been carried out for biology. Each system is applied to different datasets (text corpora), is of different scope, and is evaluated in a different way. This explains why precision/recall evaluations of text mining

Copyright © 2003 John Wiley & Sons, Ltd.

systems in this field are only comparable to a very limited extent.

### References

1. Andrade MA, Valencia A. 1998. Automatic extraction of keywords from scientific text: application to the knowledge domain of protein families. *Bioinformatics* **14**: 600–607.
2. Ashburner M, Ball CA, Blake JA, *et al*. 2000. Gene ontology: tool for the unification of biology. *Nature Genet* **25**: 25–29.
3. Blaschke C. 2002. Applications of Information Extraction Techniques to Molecular Biology. PhD Thesis, University Autónoma de Madrid, Spain.
4. Blaschke C, Oliveros JC, Valencia A. 2001. Mining functional information associated to expression arrays. *Funct Integr Genomics* **2**: 256–268.
5. Blaschke C, Valencia A. 2001. Can bibliographic pointers for known biological data be found automatically? Protein interactions as a case study. *Comp Funct Genom* **2**: 196–206.
6. Blaschke C, Valencia A. 2002. Automatic ontology construction from the literature. *Genome Inform Ser Workshop Genome*. Universal Academy Press: Tokyo, (in press).
7. Craven M, Kumlien J. 1999. Constructing biological knowledge bases by extracting information from text sources. *Proc Int Conf Intell Syst Mol Biol* (1999): 77–86.
8. Devos D, Valencia A. 2002. Intrinsic errors in genome annotation. *Trends Genet* **17**: 429–431.
9. Eisen MB, Spellman PT, Brown PO, Botstein D. 1998. Cluster analysis and display of genome-wide expression patterns. *Proc Natl Acad Sci USA* **95**: 14863–14868.
10. Franzén K, Eriksson G, Olsson F, Lidén LAP, Cöster J. 2002. Protein names and how to find them. *Int J Med Info* (in press).
11. Fukuda K, Tsunoda T, Tamura A, Takagi T. 1998. Information extraction: identifying protein names from biological papers. *Pac Symp Biocomput* (1998): 707–718.
12. Hirschman L. 1998. The evolution of evaluation: lessons from the message understanding conferences. *Comput Speech Lang* **12**: 281–305.
13. Jenssen TK, Lægreid A, Komorowski J, Hovig E. 2001. A literature network of human genes for high-throughput analysis of gene expression. *Nature Genet* **28**: 21–28.
14. Ohta Y, Yamamoto Y, Okazaki T, Uchiyama I, Takagi T. 1997. Automatic construction of knowledge base from biological papers. *Proc Int Conf Intell Syst Mol Biol* (1997): 218–225.
15. Oliveros JC, Blaschke C, Herrero J, Dopazo J, Valencia A. 2000. Expression profiles and biological function. *Genome Inform Ser Workshop Genome Inform* (2000): 106–117.
16. Perez-Iratxeta C, Bork P, Andrade MA. 2001. XplorMed: a tool for exploring MEDLINE abstracts. *Trends Biochem Sci* **26**: 573–575.
17. Proux D, Rechenmann F, Julliard L, Pillet V, Jacq B. 1998. Detecting gene symbols and names in biological texts: a first step toward pertinent information extraction. *Genome Inform Ser Workshop Genome Inform* (1998): 72–80.
18. Raychaudhuri S, Chang JT, Sutphin PD, Altman RB. 2002. Associating genes with gene ontology codes using a maximum entropy analysis of biomedical literature. *Genome Res* **12**: 203–214.
19. Shatkay H, Edwards S, Wilbur WJ, Boguski M. 2000. Genes, themes, and microarrays. Using information retrieval for large-scale gene analysis. *Proc Int Conf Intell Syst Mol Biol* (2000): 317–328.
20. Tanabe L, Wilbur JW. 2002. Tagging gene and protein names in biomedical text. *Bioinformatics* **18**: 1124–1132.
21. Wilbur WJ, Coffee L. 1994. The effectiveness of document neighboring in search enhancement. *Inf Process Manag* **30**: 253–266.
22. Wilbur WJ, Yang Y. 1996. An analysis of statistical term strength and its use in the indexing and retrieval of molecular biology texts. *Comput Biol Med* **26**: 209.

Copyright © 2003 John Wiley & Sons, Ltd. 
